# Online delivery of oral HIV pre‐ and post‐exposure prophylaxis: findings from the ePrEP Kenya pilot

**DOI:** 10.1002/jia2.26468

**Published:** 2025-06-26

**Authors:** Catherine Kiptinness, Paulami Naik, Tabitha Kareithi, Nicholas Thuo, Phelix Okello, Carlos Culquichicon, Maeve Rafferty, Samira Abdulrashid, Edwin Jomo, Nicky Nyamasyo, Tony Wood, Rouella Mendonca, Rachel C. Malen, Julia C. Dettinger, Jillian Pintye, June Mwangi, Andy Stergachis, Jonah Onentia, Kelly Curran, Melissa Latigo Mugambi, Daniel Were, Kenneth Ngure, Monisha Sharma, Katrina F. Ortblad, the ePrEP Kenya team

**Affiliations:** ^1^ Center for Clinical Research Kenya Medical Research Institute Nairobi Kenya; ^2^ Department of Global Health University of Washington Seattle Washington USA; ^3^ Department of Epidemiology University of Washington Seattle Washington USA; ^4^ Public Health Sciences Division Fred Hutchinson Cancer Center Seattle Washington USA; ^5^ MYDAWA Nairobi Kenya; ^6^ Audere Seattle Washington USA; ^7^ Department of Biobehavioral Nursing and Health Informatics Seattle Washington USA; ^8^ Jhpiego Baltimore Maryland USA; ^9^ Department of Pharmacy University of Washington Seattle Washington USA; ^10^ National AIDS Control Program Kenya Ministry of Health Nairobi Kenya; ^11^ School of Public Health Jomo Kenyatta University of Agriculture and Technology Nairobi Kenya

**Keywords:** HIV prevention, pre‐exposure prophylaxis, post‐exposure prophylaxis, online delivery, differentiated service delivery, HIV self‐testing

## Abstract

**Introduction:**

The expansion of telecommunication networks and smartphones in many African countries could be leveraged to deliver HIV prevention products directly to consumers. In collaboration with a private e‐commerce platform and online pharmacy in Kenya, MYDAWA, we piloted a new model of HIV pre‐ and post‐exposure prophylaxis (PrEP/PEP) delivery.

**Methods:**

In the ePrEP Kenya pilot (NCT05377138), individuals living in Nairobi and Mombasa Counties could complete a free telehealth visit with a remote clinician to assess eligibility for online PrEP/PEP (i.e. ≥18 years; no medical contraindications). Eligible individuals could order HIV testing services—courier delivered to clients’ choice location—for a fee of 250 KES (∼$2 USD) for self‐testing or 150 KES (∼$1 USD) for provider‐administered rapid diagnostic testing. Following confirmation of clients’ HIV‐negative status (via an uploaded test result image), free PrEP/PEP drugs from government supply were courier delivered with or separately from HIV testing services. Clients paid a delivery fee ≤149 KES (∼$1 USD) per courier visit.

**Results:**

From October 2022 to December 2023, we screened 2257 individuals and enrolled 1915. Most PrEP/PEP clients were men (63%, 1428/1915), ≥25 years (72%, 1631/1915) and never married (80%, 1796/1915); few had ever used PrEP (3%, 48/1915) or PEP (14%, 263/1915). At enrolment, 227 (12%) were preliminarily eligible for PrEP and 1688 (88%) for PEP. Among PrEP‐eligible clients, 89% (203/227) completed HIV testing and 92% (208/227) received PrEP; among PEP‐eligible clients, 92% (1551/1688) completed HIV testing and 92% (1549/1688) received PEP. Most PrEP/PEP clients completed HIV testing within 6 hours of their telehealth visit (53%, 927/1757) and had drugs delivered with testing services (88%, 1546/1757). Among PrEP clients eligible for follow‐up, 47% (120/256) continued PrEP and 4% (10/256) initiated PEP following PrEP discontinuation. Among PEP clients eligible for follow‐up, 7% (99/1428) repeated PEP use and 6% (83/1428) transitioned from PEP to PrEP.).

**Conclusions:**

Online PrEP/PEP delivery could expand access to prevention services by reaching individuals not engaged in existing delivery platforms. The uptake of online PEP was five times greater than PrEP, underscoring an unmet demand for PEP and highlighting the potential for online pharmacies to deliver time‐sensitive PEP services.

## 1 INTRODUCTION

In many African countries with high HIV incidence, e‐commerce is increasing due to expanding telecommunication networks and smartphone coverage [1, 2, 3, 4, 5]. Companies providing direct‐to‐consumer health products could be leveraged to provide HIV prevention services [5, 6]. In Kenya, ∼47 million individuals (>80% of the population) have smartphone access and there are >60 licensed, private e‐commerce companies [7]. Online delivery of HIV prevention products—including HIV self‐testing (HIVST) and pre‐ and post‐exposure prophylaxis (PrEP and PEP) drugs—has potential advantages over clinic delivery, including increased convenience and privacy [8, 9, 10, 11], and could expand prevention services to eligible populations unrepresented in clinics (e.g. men, young people) [12]. When paired with telemedicine—which was effectively used in Kenya to maintain HIV service access during COVID‐19 [13, 14]—e‐commerce platforms could operate as one‐stop shops for HIV service delivery.

Kenya, a leader in differentiated models of HIV service delivery [15], is an ideal setting to evaluate an online PrEP/PEP delivery model. In Kenya, most HIV acquisition occurs in urban areas [16] where there is a growing middle class [17] with access to technology and the ability to pay for products delivered via e‐commerce platforms [18]. In 2022, Kenya identified private pharmacies as a target PrEP delivery platform [19] and, in 2023, established a private‐sector framework for HIV service delivery [20]. Pilot studies of PrEP/PEP delivery at private, brick‐and‐mortar pharmacies in Kenya have demonstrated feasibility and high uptake among populations underserved at public clinics (i.e. unmarried individuals) [21], but no study to our knowledge has evaluated PrEP/PEP delivery via a private online pharmacy in the region.

Online pharmacies may be particularly well‐suited to deliver PEP, which is most effective ≤24 hours (and up to 72 hours) of HIV exposure [22]. Compared to clinics and some brick‐and‐mortar pharmacies, online pharmacies have longer operating hours (including evenings and weekends) and can deliver discreet services quickly to clients’ preferred settings, enabling clients to more easily initiate PEP within the recommended window [22]. While Kenya has recommended PEP use for all individuals with recent HIV exposure since 2016 [23], its use has not been widely promoted and access remains limited beyond cases of occupational exposure or sexual assault [23, 24, 25]. Bias among healthcare providers reporting moral conflicts also limits PEP provision [26].

To understand the potential for private online pharmacies to reach eligible individuals and deliver public HIV commodities, we partnered with MYDAWA [27], an online pharmacy and e‐commerce platform in Kenya, to develop and evaluate the feasibility, uptake and acceptability of a novel online PrEP/PEP delivery model [28].

## 2 METHODS

### 2.1 Study design and setting

We conducted a single‐arm, prospective pilot study of online oral PrEP/PEP delivery in Nairobi and Mombasa Counties (the latter was introduced 8 months into implementation). In 2022, these counties reported >2500 HIV incident cases, with Nairobi reporting 1999 cases—the most of any Kenyan county [16].

MYDAWA, established in 2017, is an e‐commerce platform delivering prescription and non‐prescription drugs and other products (e.g. shampoo, diapers) to clients in Nairobi and Mombasa Counties. In 2023, MYDAWA had ∼68,000 clients and sexual and reproductive health (SRH) products comprised ∼21% of sales [29]. To enable the delivery of prescription medication, MYDAWA is licensed as both an online pharmacy and medical facility; the latter of which occurred just prior to study implementation, enabling PrEP/PEP prescribing by MYDAWA clinicians via telehealth. During pilot implementation, MYDAWA telehealth visits were available from 8 AM to 10 PM, and orders placed from 8 AM to 7 PM had guaranteed delivery within 6 hours.

### 2.2 Care pathway

In collaboration with PrEP implementors, researchers and MYDAWA leadership, we developed an online PrEP/PEP delivery care pathway adapted from one for brick‐and‐mortar pharmacy PrEP delivery in Kenya (Figure S1) [28, 30]. Prior to implementation, PrEP/PEP implementors and Kenya Ministry of Health trainers conducted a 3‐day training for MYDAWA clinicians and pharmaceutical technologists (“pharm techs”) that followed the national PrEP/PEP curriculum [31] and covered the pathway's core components: behavioural HIV risk and medical safety assessments—delivered by clinicians via telehealth visits—and HIV testing and drug dispensing—delivered by pharm techs via courier visits. Pharm techs completed practicum sessions on HIV testing in clinics. PrEP/PEP implementors provided technical assistance bi‐monthly and consultations as needed.

### 2.3 Participants

Eligible participants were ≥18 years old and met the prescribing checklist criteria (described below). To generate demand for online PrEP/PEP, MYDAWA created a “Gen‐N community” marketing campaign—with Gen‐N referencing an HIV‐negative generation—to motivate individuals to engage in HIV prevention services and contribute to an AIDS‐free future (Figure S2). MYDAWA marketed online PrEP/PEP via social media (e.g. Facebook, Instagram), search engines (e.g. Google) and their website using banners, pop‐ups, stickers and landing pages that targeted clients purchasing SRH products. During pilot implementation, MYDAWA was one of the top Google hits for individuals searching “PrEP” or “PEP” in Nairobi. Additionally, MYDAWA hosted four in‐person events between February and June 2023 to promote online PrEP/PEP on university campuses and in Nairobi neighbourhoods.

The study protocol was approved by the Scientific Ethics Review Unit at Kenya Medical Research Institute. Participants completed electronic informed consent (via email or SMS) during their initial telehealth visit and received 500 Kenyan Shillings (KES; ∼$3.50 US Dollars [USD]) for completing behavioural surveys.

### 2.4 Study procedures

#### 2.4.1 Telehealth visits

We worked with ClickMedix (Cambridge, USA)—a global mobile health social enterprise [32]—to integrate a secure telehealth portal into MYDAWA's platform. Interested clients could schedule free telehealth visits via MYDAWA's website.

At telehealth visits, clients provided their names and phone numbers; MYDAWA clinicians then utilized a prescribing checklist (Figure S3) to determine clients’ online PrEP or PEP eligibility and guide counselling on the appropriate service. First, clinicans screened clients for HIV acquisition risk using a modified version of Kenya's HIV Risk Assessment Screening Tool (RAST) [28], which asked about behaviours in the past 6 months (e.g. partners of unknown HIV status, transactional sex) and potential HIV exposures in the past 72 hours (condomless sex with someone who may have HIV; sexual assault; and exposure to blood/other bodily fluids). MYDAWA clinicians classified clients as potentially eligible for either PrEP or PEP based on clients’ RAST responses, HIV risk perception and product preferences. Next, providers screened potential clients for symptoms of acute HIV acquisition and PrEP clients for medical conditions (e.g. history of liver or kidney disease) that might contraindicate PrEP safety. Serum creatine and hepatitis B/C testing were not conducted, as national guidelines state that the availability of these tests should not delay PrEP/PEP initiation [22].

Clients who met the checklist criteria for online PrEP/PEP received a conditional prescription (pending confirmation of HIV‐negative status) and those who did not were referred to nearby clinics. To continue online PrEP, clients scheduled a free follow‐up telehealth visit and repeated the prior steps, plus screening for potential drug side effects. Online PEP clients were encouraged to complete a follow‐up telehealth visit 28 days post‐initiation for repeat HIV testing and PrEP counselling.

#### 2.4.2 Courier delivery

Following telehealth visits, eligible clients could order HIV testing services and PrEP/PEP via MYDAWA and have these courier‐delivered to their preferred location by a pharm tech on a motorcycle. Clients had two HIV testing options: (1) HIVST, for a subsidized fee of 250 KES (∼$2 USD), or (2) provider‐administered rapid diagnostic testing (RDT) by a pharm tech, for a non‐subsidized fee of 150 KES (∼$1 USD); the latter was introduced 8 months into implementation. Additionally, clients could choose to have HIV testing and PrEP/PEP delivered in the same (one‐step) or separate (two‐step) courier visits. Since we used government PrEP/PEP commodities, we did not charge clients for drugs; however, a delivery fee of up to 149 KES (∼$1 USD) was applied per courier visit.

Strategies to confirm clients’ HIV‐negative status prior to PrEP/PEP dispensing varied by HIV test type. For HIVST, MYDAWA clinicians reviewed test result images uploaded to the online platform. We collaborated with Audere (Seattle, USA)—a digital health non‐profit [33]—to incorporate an artificial intelligence (AI) algorithm into the platform that prompted clients to upload a new HIVST result image if the previous one was likely uninterpretable (e.g. was blurry, did not include the results window). For RDT, pharm techs interpreted and communicated test results to MYDAWA clinicians. Clients who tested HIV positive received post‐test counselling from a MYDAWA clinician (via a scheduled telehealth visit) or pharm tech and were referred to nearby clinics for confirmatory testing and treatment.

After MYDAWA clinicians confirmed clients’ HIV‐negative status, pharm techs were approved (often via phone calls) to deliver oral PrEP/PEP per national guidelines [34]; clients initiating PrEP or PEP received a 30‐ or 28‐day drug supply, respectively, and those refilling PrEP received a 90‐day supply. Clients were advised to use oral PrEP daily and not counselled on event‐driven PrEP, which was not included in national guidelines at the time.

### 2.5 Data collection and management

We obtained data on all clients from MYDAWA pharmacy records and select clients from behavioural surveys, which all clients were invited to complete within 2 weeks of enrolment. From pharmacy records, we obtained clients’ demographics, behaviours associated with HIV acquisition, HIV testing results, timing of their potential HIV exposure (PEP clients only), and date/time of their telehealth and courier visits (recorded by pharm techs following delivery). In behavioural surveys, we captured additional demographics, sexual behaviours, PrEP/PEP knowledge/prior use and perceptions of online PrEP/PEP delivery.

### 2.6 Outcomes

Primary outcomes were PrEP and PEP initiation (any dispensing) and PrEP continuation (any refills) within 45 days of initiation. Additionally, we measured any PrEP continuation over the observation period and at scheduled follow‐up visits; clients who refilled PrEP ≤15 days from a scheduled visit were considered “on‐time” and those who refilled >15 days from a scheduled visit were considered to have “stopped and restarted” [35]. Secondary outcomes included PEP initiation following PrEP discontinuation, PEP‐to‐PrEP transition and repeated PEP use. Process outcomes included day/time and duration of telehealth visits, client selection of HIV test type and one‐ versus two‐step delivery, information on HIVST images uploaded and the time between delivery steps.

Implementation outcomes [28] included clients’ perceived acceptability, satisfaction and willingness to pay for various delivery steps (delivery fees excluded). Additionally, we assessed clients’ experiences and perceptions of service quality. We measured acceptability and satisfaction using statements, with 5‐point Likert scale responses, that assessed different components of the Theoretical Framework of Acceptability [36] and Client Satisfaction Questionnaire [37].

### 2.7 Analyses

We used descriptive statistics for most outcomes, adjusting the sample to those eligible (based on the service dispensed or clients’ follow‐up duration). For continuation outcomes, we conducted subgroup analyses for: men ≥25, women ≥25, men <25 and women <25 years. For factors associated with any PrEP continuation (among PrEP clients), and repeated PEP use and PEP‐to‐PrEP transition (among PEP clients), we utilized negative binomial regression models with robust standard errors adjusted for a priori variables. For several implementation outcomes, we aggregated the two most positive response categories and considered a construct achieved if ≥80% of responses were in this aggregated category [38]. We conducted analyses in R (v6.1) [39].

## 3 RESULTS

**Figure 1:**
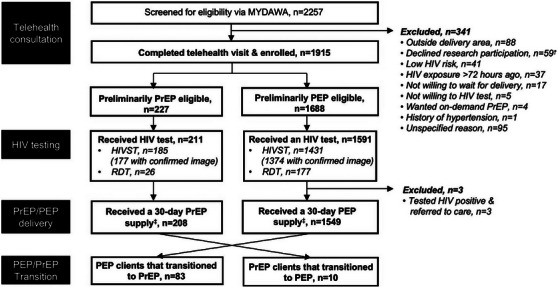
**Flow of participants through the care pathway for online PrEP/PEP delivery**. Abbreviations: HIVST, HIV self‐testing; PEP, post‐exposure prophylaxis; PrEP, pre‐exposure prophylaxis. ^†^Thirty‐three clients (6 PrEP; 27 PEP clients) received medicine from MYDAWA without participating in the study (i.e. paying the full price of PrEP/PEP after seeing a MYDAWA clinician in person or uploading a prescription from another facility). ^‡^Thirty‐five clients (5 PrEP; 30 PEP) clients received PrEP/PEP delivery without having uploaded an image of their HIV test (due to clinician error).

Between October 2022 and December 2023, 2257 individuals began a telehealth visit and 1915 enrolled in the study (Figure 1). Common exclusion reasons included living outside delivery area (26%, 88/341) and declining research participation (17%, 59/341). Among individuals enrolled, 227 (12%) were determined preliminarily eligible for PrEP and 1688 (88%) for PEP. Most clients learned of online PrEP/PEP from the MYDAWA website (45%, 867/1915), followed by Google ads (32%, 615/1915) (Table 1). At enrolment, 38% (736/1915) of eligible clients agreed to participate in behavioural surveys—41% (93/227) of PrEP and 38% (638/1688) of PEP clients; the characteristics of clients who did and did not participate in behavioural surveys were largely similar (Table S4).

**Table 1:** Characteristics of online PrEP/PEP clients who enrolled, initiated services and completed behavioural surveys

	Preliminarily eligible online PrEP/PEP clients		Clients who initiated online PrEP/PEP		Clients who completed behavioural surveys^a^	
Characteristic	PrEP (*n* = 227)	PEP (*n* = 1688)	PrEP (*n* = 208)	PEP (*n* = 1549)	PrEP (*n* = 93)	PEP (*n* = 638)
**Demographics**						
Age: Median [IQR]	28 [24, 34]	27 [24, 33]	28 [25, 34]	27 [24, 33]	26 [19, 56]	27 [24, 31]
*Age ≥ 25 years*	162 (71%)	1222 (72%)	154 (74%)	1119 (72%)	59 (63%)	439 (69%)
Sex: *Male* ^b^	169 (74%)	1066 (63%)	155 (75%)	966 (62%)	57 (61%)	396 (62%)
Married	26 (12%)	212 (13%)	24 (12%)	196 (13%)	5 (5%)	78 (12%)
Relationship status^c^						
*Casual partners only*	−	−	−	−	45 (48%)	319 (50%)
*Primary partner only*	−	−	−	−	29 (31%)	220 (34%)
*Primary and casual partners*	−	−	−	−	19 (20%)	71 (11%)
Occupation						
*Professional* ^d^	−	−	−	−	40 (43%)	306 (48%)
*Student*	−	−	−	−	20 (22%)	50 (8%)
*Trade/Sales*	−	−	−	−	15 (16%)	140 (22%)
*None*	−	−	−	−	14 (15%)	91 (14%)
*Other*	−	−	−	−	4 (4%)	49 (8%)
Monthly income (KES) [USD]^e^						
*≤10,000* [≤$70]	−	−	−	−	32 (34%)	150 (23%)
*10,001−70,000* [$71−$500]	−	−	−	−	32 (34%)	322 (51%)
*70,001−150,000* [$501−$1070]	−	−	−	−	16 (16%)	109 (17%)
*>150,000* [>$1070]	−	−	−	−	13 (14%)	52 (8%)
Special populations						
*Men who have sex with men (/men)*	47 (28%)	28 (3%)	45 (29%)	26 (3%)	15 (16%)	15 (2%)
*Member of an HIV serodiscordant couple*	10 (4%)	1 (0·1%)	10 (5%)	1 (0·1%)	3 (3%)	0 (%)
**Health history** ^f^						
Currently using PrEP	22 (10%)	0 (0%)	22 (11%)	0 (0%)	6 (7%)	1 (0.2%)
Prior PrEP use	36 (16%)	12 (1%)	35 (17%)	12 (1%)	13 (14%)	6 (1%)
Prior PEP use	36 (16%)	227 (13%)	30 (14%)	211 (14%)	15 (16%)	100 (16%)
Prior emergency contraception use ≥2 times	−	−	−	−	32 (34%)	289 (45%)
Currently using LARC (/women)	−	−	−	−	11 (12%)	81 (33%)
Pregnant/breastfeeding (/women)	3 (1%)	23 (4%)	3 (1%)	23 (1%)	2 (6%)	9 (3%)
Prior pregnancy (/women)	−	−	−	−	18 (53%)	103 (42%)
**Behaviours associated with risk of HIV acquisition** ^f^						
In the past 6 months						
*Multiple concurrent sexual partners*	144 (63%)	789 (47%)	136 (65%)	716 (46%)	56 (60%)	284 (44%)
*Partner(s) of unknown HIV status*	170 (75%)	1507 (89%)	153 (74%)	1384 (89%)	72 (78%)	573 (89%)
*Partner(s) living with HIV*	25 (11%)	43 (3%)	24 (12%)	42 (3%)	9 (10%)	16 (3%)
*Inconsistent condom use*	112 (49%)	1231 (73%)	103 (50%)	1143 (74%)	45 (48%)	455 (71%)
*Transactional sex*	6 (3%)	45 (3%)	6 (3%)	41 (3%)	2 (2%)	21 (3%)
*STI diagnosis*	7 (3%)	47 (3%)	6 (3%)	41 (3%)	4 (4%)	20 (3%)
*Needle sharing for drug use*	0 (0%)	4 (0%)	0 (0%)	3 (0%)	0 (0%)	0 (0%)
*Forced sex/sexual assault*	5 (2%)	38 (2%)	3 (1%)	37 (2%)	1 (1%)	16 (3%)
*Used PEP 2+ times*	13 (6%)	62 (4%)	9 (4%)	61 (4%)	5 (5%)	24 (4%)
In the past 72 hours^f^						
*Condomless sex and potential risk of HIV acquisition*	7 (3%)	1201 (71%)	6 (3%)	1123 (73%)	5 (5%)	470 (73%)
*Sexual assault*	0 (0%)	17 (1%)	0 (0%)	15 (1%)	0 (0%)	3 (0.5%)
*Exposure to bodily fluids: non‐sexual*	0 (0%)	70 (4%)	0 (0%)	62 (4%)	0 (0%)	23 (4%)
*Exposure to bodily fluids: sexual*	1 (0.4%)	360 (21%)	1 (0.5%)	316 (20%)	1 (1%)	138 (22%)
Self‐assessment of HIV risk in the next month						
*High*	81 (36%)	225 (13%)	75 (36%)	204 (13%)	23 (25%)	81 (13%)
*Medium*	103 (45%)	1027 (61%)	96 (46%)	954 (62%)	48 (52%)	403 (63%)
*Low*	42 (19%)	428 (25%)	36 (17%)	387 (25%)	22 (24%)	152 (24%)
**Knowledge of online PrEP/PEP**						
How potential clients heard about online PrEP/PEP^f^						
*MYDAWA website*	86 (38%)	781 (46%)	84 (40%)	726 (47%)	40 (43%)	308 (48%)
*Google ads*	41 (18%)	574 (34%)	38 (18%)	535 (35%)	10 (11%)	219 (34%)
*Social media*	40 (18%)	169 (10%)	30 (14%)	149 (10%)	14 (15%)	67 (11%)
*Peer/friend/sex partner*	33 (15%)	110 (7%)	32 (15%)	96 (6%)	10 (11%)	41 (6%)
*Healthcare provider*	6 (3%)	62 (4%)	5 (2%)	55 (4%)	4 (4%)	14 (2%)
*Campus event*	28 (12%)	11 (1%)	25 (12%)	11 (1%)	20 (22%)	7 (1%)

Abbreviations: KES, Kenyan Shilling; LARC, long‐acting reversible contraception; PEP, post‐exposure prophylaxis; PrEP, pre‐exposure prophylaxis; USD, United States Dollar.

^a^ All enrolled individuals were invited to complete a behavioural survey 2 weeks after enrolling as a PrEP participant and 1.5 months after enrolling as a PEP participant; 41% (93/227) of individuals eligible for PrEP and 38% (638/1688) of individuals eligible for PEP agreed to participate.

^b^ This includes two PrEP clients who identified as intersex (included in all PrEP categories) and one PEP client who identified as intersex; this individual did not participate in the behavioural survey.

^c^ Among PEP clients, 33 (5%) chose not to answer the relationship status question and seven (1%) chose not to answer the monthly income question.

^d^ Professionals included medical officers, lawyers, teachers, graphics designers and accountants.

^e^ Average USD/KSH 2023 exchange rate during the implementation period was 140 KSH per 1 USD.

^f^ Categories are not mutually exclusive.

### 3.1 PrEP/PEP initiation

HIV testing uptake among preliminarily eligible online PrEP/PEP clients (including uploading HIVST test results) was 92% (1754/1915), and similar for PrEP and PEP clients (Figure 1). Three clients tested HIV positive prior to PrEP/PEP initiation; all were preliminarily PEP eligible. PrEP initiation among eligible clients was 92% (208/227), as was PEP initiation (92%, 1549/1688). A few PrEP/PEP clients (2%, 35/1688) were dispensed drugs without confirmed HIV testing results due to implementation errors; all were followed up and confirmed HIV negative. A few PrEP clients (*n* = 10) discontinued PrEP and later initiated PEP and a few PEP clients transitioned to PrEP (*n* = 83), resulting in 291 unique PrEP and 1559 unique PEP clients.

Table 1 shows the demographics of clients who enrolled in the study, initiated online PrEP/PEP and completed behavioural surveys. Among clients who initiated PrEP/PEP, most were ≥25 years (72%; 1273/1757), male (64%; 1121/1757) and unmarried (87%, 1537/1757). While few clients identified as members of priority populations, more male PrEP clients (29%, 45/155) reported sex with men than male PEP clients (3%, 26/966; *p*<0.01). While prior PrEP or PEP use was low among all clients, prior PrEP use was significantly higher among PrEP (17%, 35/208) compared to PEP clients (1%, 12/1549; *p*<0.01).

Prevalence of behaviours associated with HIV acquisition was high among those eligible for online PrEP/PEP; 87% (1537/1757) reported sexual partners with unknown HIV status and 48% (852/1757) reported multiple concurrent sexual partners in the past 6 months. Compared to eligible PEP clients, significantly more eligible PrEP clients reported multiple concurrent sexual partners (63%, 144/227 vs. 47%, 789/1688; *p*<0.01) and high self‐perceived HIV acquisition risk (36%, 81/227 vs. 13%, 225/1688; *p*<0.01). Most eligible PEP clients reported condomless sex in ≤72 hours with someone who might have HIV (71%, 1201/1688); few reported a recent sexual assault (1%, 17/1688).

### 3.2 PrEP continuation and repeat PEP use

**Figure 2:**
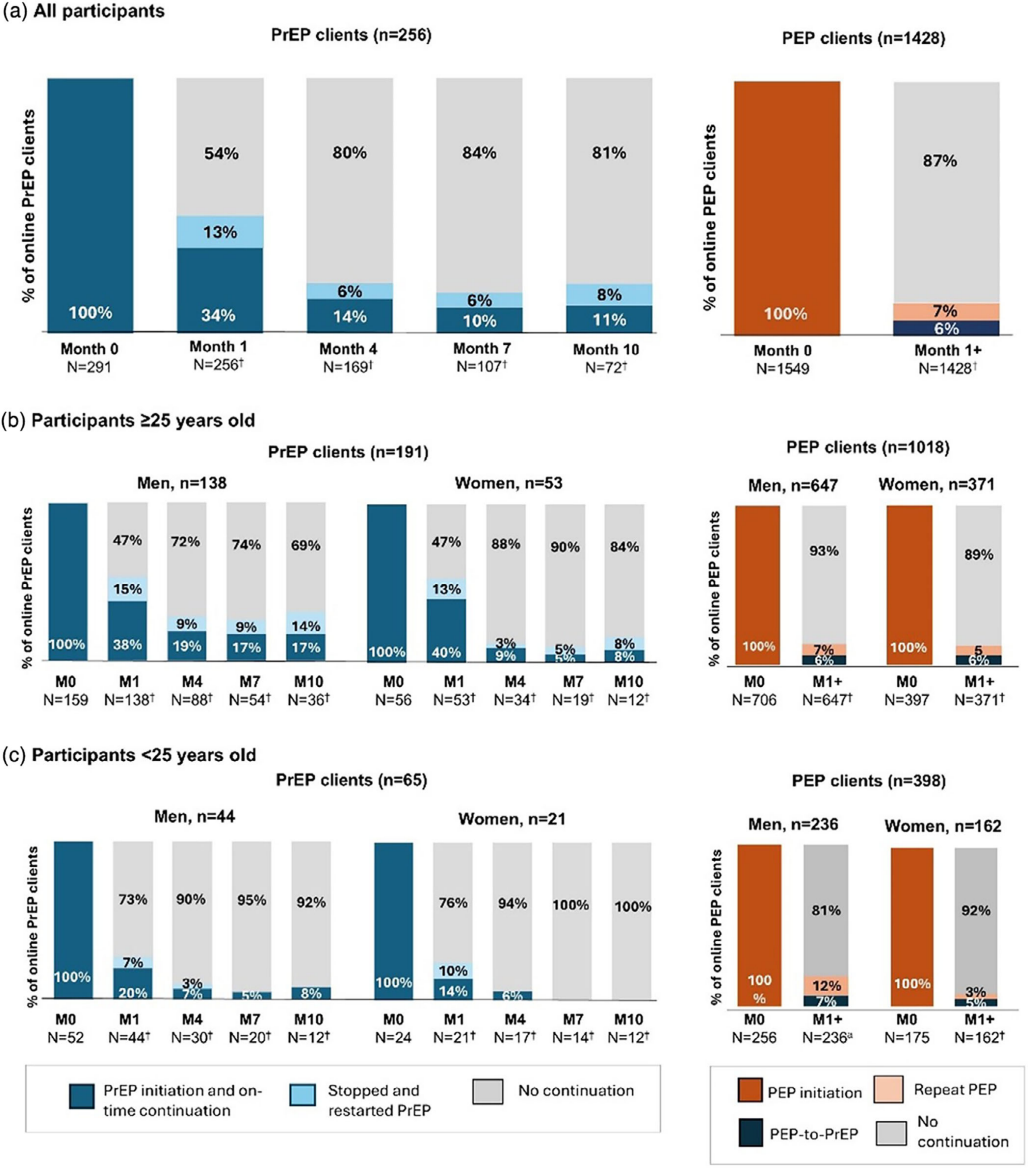
**Online PrEP and PEP continuation over the pilot duration among eligible clients who initiated services, by age and sex**. Abbreviations: M, month; PEP, post‐exposure prophylaxis; PrEP, pre‐exposure prophylaxis. ^†^Among those initiated, enrolled and eligible for a follow‐up visit.

We observed 9746 client months of follow‐up: 1212 months for PrEP clients and 8534 months for PEP clients. Any PrEP continuation among clients eligible for follow‐up (i.e. >1 month from initiation) was 34% (88/256) by 45 days and 47% (120/256) over the observation period (Figure 2). At 7 months post PrEP initiation, 11% (12/108) of eligible clients were still engaged in online PrEP services. Any PrEP continuation over pilot duration was similar among men and women, but significantly higher among those ≥25 years (53%, 102/191) compared to those <25 years (28%, 18/65; *p*<0.01). PEP initiation following PrEP discontinuation among eligible clients was 4% (10/256). At 28 days post PEP initiation, 18% (259/1428) of eligible clients completed repeat HIV testing and 16% (227/1428) completed a follow‐up telehealth visit (median time from initiation: 34 days, IQR 30–53 days). Repeat PEP use among eligible clients was 7% (99/1428); men <25 years were significantly more likely to repeat PEP than other subgroups (12%, 29/236 vs. 6%, 70/1192; *p*<0.01). Transition from PEP to PrEP among eligible clients was 6% (83/1428). No online PrEP/PEP clients tested HIV positive at follow‐up.

In adjusted models among PrEP clients, variables associated with higher likelihood of PrEP continuation were: age ≥25 years, males reporting sex with men and having a partner living with HIV (Table 2). Prior PEP use was associated with a 36% lower likelihood of PrEP continuation although it did not reach statistical significance (*p* = 0.09). Among PEP clients, variables associated with a higher likelihood of PEP‐to‐PrEP transition were: men reporting sex with men, prior PrEP use and prior PEP use; being married was associated with a 90% lower likelihood of PEP‐to‐PrEP transition (*p* = 0.02). Among PEP clients, a higher likelihood of repeat PEP use was associated with: being male and reporting prior PEP use. For unadjusted models, see Tables S1 and S2.

**Table 2:** Characteristics associated with PrEP continuation, PEP‐to‐PrEP transition and repeat PEP use, findings from adjusted multivariable models

	Initiated PrEP (*n* = 256)^a^				Initiated PEP (*n* = 1428)^b^							
	PrEP continuation:				PEP‐to‐PrEP transition:				Repeat PEP use:			
Characteristic	PrEP refills (*n* = 88)	No PrEP refills (*n* = 168)	aRR (95% CI)	*p*	PrEP transition (*n* = 82)	No PrEP transition (*n* = 1346)	aRR (95% CI)	*p*	Repeat PEP (*n* = 99)	No repeat PEP (*n* = 1329)	aRR (95% CI)	*p*
**Demographics**												
*Age ≥25 years*	**75** **(85%)**	**114** **(69** **%)**	**1.80** **(1.04−2.99)**	**0.03**	57 (70%)	962 (72%)	1.07 (0.67−1.71)	0.77	66 (67%)	953 (73%)	0.75 (0.50‐1.12)	0.16
*Sex: Male*	64 (73%)	118 (70%)	0.88 (0.60−1.30)	0.52	53 (65%)	838 (62%)	1.04 (0.65−1.66)	0.86	**75** **(76%)**	**816** **(62%)**	**1.77** **(1.13‐2.79)**	**0.01**
*Married*	−	−	−	−	**1** **(1%)**	**174** **(13%)**	**0.10** **(0.01−0.73)**	**0.02**	−	−	–	–
*Men who have sex with men*	**21 (24%)**	**21** **(13%)**	**1.74** **(1.16−2.60)**	**0.01**	**8** **(10%)**	**16** **(1%)**	**4.25** **(2.15−8.40)**	**<0.01**	−	−	–	–
**Health history**												
*Prior PrEP use* ^c^	−	−	−	−	**3** **(4%)**	**8** **(1%)**	**2.96** **(1.07−8.18)**	**0.04**	−	−	–	–
*Prior PEP use* ^c^	14 (16%)	45 (27%)	0.64 (0.39−1.16)	0.09	**20** **(25%)**	**175** **(13%)**	**1.85** **(1.13−3.01)**	**0.01**	**26** **(26%)**	**169** **(13%)**	**2.07** **(135‐3.18)**	**<0.01**
*STI diagnosis*	−	−	−	−	5 (6%)	35 (3%)	1.83	0.22				
**Sexual behaviours**												
*1+ sex partner*	58 (66%)	93 (56%)	1.25 (0.87−1.78)	0.23	−	−	−	−	−	−	‐	–
*Partner living with HIV*	**12 (14%)**	**9** **(5%)**	**2.00** **(1.29−3.10)**	**<0.01**		−	−	−	5 (5%)	34 (3%)	1.66 (0.70‐3.94)	0.25

Abbreviations: aRR, adjusted risk ratio; PEP, post‐exposure prophylaxis; PrEP, pre‐exposure prophylaxis; STI, sexually transmitted infections. Bolded values indicate those that met our significance threshold of p<0.05.

^a^ Included clients who initially initiated PrEP or transitioned from PEP to PrEP at least 30 days before the follow‐up period ended. Excludes clients who initiated PrEP <1 month from pilot completion and thus were not eligible for follow‐up.

^b^ Included clients who initially initiated PEP at least 28 days before the follow‐up period ended. Excludes clients who initiated PEP < 28 days from pilot completion and thus were not eligible for follow‐up.

^c^ Excludes clients who initiated PEP <1 month from pilot completion and thus were not eligible for follow‐up.

### 3.3 Process outcomes

We observed 2608 telehealth visits (including initiation and continuation visits): 548 for PrEP and 2060 for PEP clients. On average, telehealth visits lasted 16 minutes (IQR 13–18 minutes), with PrEP/PEP initiation visits taking slightly longer than PrEP continuation visits (Table 3). Most telehealth visits occurred either on Mondays (17%, 455/2608) or Tuesdays (16%, 426/2608); 26% (670/2608) occurred after 5 PM and 26% (669/2608) on the weekends.

**Table 3:** Process outcomes associated with online PrEP and PEP delivery

Process outcome	Eligible online PrEP clients (*n* = 227)	Eligible online PEP clients (*n* = 1688)
**Telehealth consultation**		
Duration (in minutes) of initial visit: median [IQR]	17 [15, 20]	15 [13, 18]
Duration (in minutes) of follow‐up visit: median [IQR]	12 [10, 15]	−
Day of the week: (/total telehealth visits^a^)		
*Sunday*	42/548 (8%)	290/2060 (14%)
*Monday*	68/548 (12%)	387/2060 (19%)
*Tuesday*	94/548 (17%)	332/2060 (16%)
*Wednesday*	93/548 (17%)	274/2060 (13%)
*Thursday*	87/548 (16%)	286/2060 (14%)
*Friday*	99/548 (18%)	219/2060 (11%)
*Saturday*	65/548 (12%)	272/2060 (13%)
Time of the day: (/total telehealth visits^a^)		
*Clinic hours: 8 AM−5 PM*	421/548 (77%)	1517/2060 (74%)
*After clinic hours: 5 PM−10 PM*	127/548 (23%)	543/2060 (26%)
**HIV testing services**		
Received HIVST versus provider rapid diagnostic testing	202/227 (89%)	1532/1688 (91%)
*Uploaded image of HIVST result (/HIVST clients)*	168/202 (83%)	1341/1532 (88%)
*Upload image of an HIVST result 1+ time (/HIVST clients)*	66/202 (33%)	275/1532 (18%)
*AI prompted image of an HIVST result to be reuploaded (/HIVST clients)*	24/202 (11%)	112/1532 (7%)
*Uploaded image of an unsupported HIVST type (/HIVST clients)*	1/202 (1%)	7/1532 (1%)
*Uploaded image of HIVST result uninterpretable* ^b^	2/202 (1%)	10/1532 (1%)
**PrEP/PEP delivery**		
Two‐step delivery: received PrEP/PEP separate from HIV testing	66/227 (29%)	275/1688 (16%)
Elected to pick‐up PrEP/PEP at MYDAWA offices (vs. courier delivery)	22/227 (10%)	140/1688 (8%)
Received PEP within 72 hours of potential exposure^c^	−	629/653 (96%)
**Hours between delivery steps and potential HIV exposure**		
Telehealth visit → HIV test result upload: median [IQR]	5 [0.5, 23]	5 [3, 15]
HIV test upload→ PrEP/PEP delivery: median [IQR] (/two‐step delivery)	5 [2, 20]	3.5 [2, 9]
Telehealth visit→ PrEP/PEP delivery: median [IQR]	5.5 [3, 18]	5 [3, 12]
Potential HIV exposure → Telehealth visit: median [IQR]^c^		26 [18, 44]
Potential HIV exposure → PEP delivery: median [IQR]^c^	−	39 [25, 51]

Abbreviations: AI, artificial intelligence; HIVST, HIV self‐testing; IQR, interquartile range; PEP, post‐exposure prophylaxis; PrEP, pre‐exposure prophylaxis.

^a^ Includes 2608 total telehealth visits over the pilot duration, 548 PrEP initiation and continuation visits and 2060 PEP initiation and repeat use visits.

^b^ Test images deemed uninterpretable for reasons including: blood smear or object obstructing view of test and control lines; blurry image.

^c^ Among PEP clients who reported the timing of their potential HIV exposure in their initial telehealth visit.

Most eligible clients (91%, 1734/1915) chose HIVST over RDT. Among clients receiving HIVST, most uploaded an image of an HIVST result (87%, 1509/1735) with few issues; the AI feature only prompted 8% (136/1734) of clients to re‐upload an HIVST image. Few clients (18%, 341/1915) chose two‐ versus one‐step delivery of HIV testing and PrEP/PEP and significantly fewer PEP clients (16%, 275/1688) compared to PrEP clients (29%, 66/227; *p*<0.01) selected this option. Overall, clients received services quickly, with a median of 5 hours (IQR 3–16) between telehealth visit and PrEP/PEP delivery. Most PEP clients (96%, 629/653) received PEP within 72 hours of their HIV exposure; the median time from reported exposures to delivery was 26 hours (IQR 18–44).

### 3.4 Implementation outcomes

Clients who completed behavioural surveys perceived online PrEP/PEP delivery as acceptable, satisfactory and high‐quality; most reported a willingness to pay for some or all model components (Table 4) (see Table S3 for details). Most clients (>90%) liked the model, were confident in their ability to access it and thought it could help prevent HIV spread in their community. Most clients (>93%) reported that MYDAWA providers were easy to understand, encouraged questions and were respectful. Many clients (>83%) reported “extremely positive” or “positive” experiences across the model components.

**Table 4:** Implementation outcomes associated with online PrEP/PEP service delivery, as assessed in behavioural surveys^a^

Implementation outcomes	Outcome^b^	Online PrEP clients, *n* = 93	Online PEP clients, *n* = 638
**Acceptability**: Assessed with the TFA; 5‐point Likert scale responses^c^			
*Liked getting PrEP/PEP online (TFA: affective attitude)*	Completely agree/agree	91 (98%)	614 (96%)
*Took a lot of effort to get PrEP/PEP online (TFA: burden)*	Completely disagree/disagree	70 (75%)	484 (75%)
*Confident in ability to get PrEP/PEP online (TFA: self‐efficacy)*	Completely agree/agree	87 (94%)	620 (96%)
*Getting PrEP/PEP online can help prevent the spread of HIV (TFA: perceived effectiveness)*	Completely agree/agree	84 (90%)	572 (89%)
*Getting PrEP/PEP online was acceptable*	Completely agree/agree	89 (96%)	620 (96%)
**Satisfaction**: Assess with the CSQ‐8; 8−32 total points.^d^ Mean (SD)	8−32 points	31.6 (1.03)	31.4 (1.32)
*Quality of the online PrEP/PEP services received*	1−4 points	3.91 (0.29)	3.82 (0.42)
*Got the online PrEP/PEP services wanted*	1−4 points	3.96 (0.33)	3.97 (0.22)
*The online PrEP/PEP programme met their needs*	1−4 points	3.85 (0.39)	3.85 (0.37)
*Would recommend online PrEP/PEP services to a friend in need*	1−4 points	3.98 (0.15)	3.98 (0.17)
*Satisfied with the amount of help received with online PrEP/PEP*	1−4 points	3.97 (0.18)	3.95 (0.30)
*Online PrEP/PEP helped addressed HIV prevention concerns*	1−4 points	3.97 (0.18)	3.97 (0.20)
*General satisfaction with online PrEP/PEP services received*	1−4 points	3.95 (0.23)	3.93 (0.31)
*Would seek online PrEP/PEP services again, if needed*	1−4 points	3.97 (0.23)	3.95 (0.30)
**Experiences with the intervention**: 5‐point Likert scale responses			
*Learning about PrEP/PEP on MYDAWA's “My Health Center” page*	Extremely positive/positive	78 (84%)	550 (87%)
*Consulting remote clinician for PrEP/PEP prescription*	Extremely positive/positive	88 (95%)	617 (96%)
*Ordering HIVST from MYDAWA* ^e^ *(/HIVST users)*	Extremely positive/positive	72 (77%)	552 (86%)
*Getting HIVST delivered from MYDAWA* ^e^ *(/HIVST users)*	Extremely positive/positive	73 (79%)	542 (84%)
*Uploading HIVST result image to MYDAWA* ^e^ *(/HIVST users)*	Extremely positive/positive	73 (79%)	492 (77%)
*Having RDT administered by a MYDAWA provider* ^e^ *(/RDT users)*	Extremely positive/positive	6 (100%)	49 (100%)
*Getting PrEP/PEP delivered from MYDAWA*	Extremely positive/positive	81 (87%)	593 (92%)
**Quality of care received**: 5‐point Likert scale responses			
*Call did not drop during remote consultation*	−	77 (83%)	585 (91%)
*Connection for remote consultation was stable*	Strongly agree/agree	83 (89%)	608 (95%)
*MYDAWA clinician used language that was easy to understand*	Strongly agree/agree	92 (99%)	631 (98%)
*MYDAWA clinician acted judgemental*	Strongly disagree/disagree	89 (96%)	601 (94%)
*MYDAWA clinician encouraged questions*	Strongly agree/agree	90 (97%)	626 (97%)
*MYDAWA clinician was respectful*	Strongly agree/agree	92 (99%)	631 (98%)
*MYDAWA clinician listened without interrupting*	Strongly agree/agree	93 (100%)	629 (98%)
*Participant willing to seek help from same MYDAWA clinician again*	Strongly agree/agree	87 (94%)	629 (98%)
**Willingness to pay for online**: *n* (%); Prices in USD, median (IQR)^f^			
*Telehealth visit*	−	76 (76%); $3.57 ($1.43−$5.18)	510 (80%); *$3.57 ($2.14 ‐ $5.00)*
*Blood‐based HIVST*	−	94 (94%); $1.79 ($1.43−$2.14)	581 (91%); $1.79 ($1.43 ‐ $2.14)
*PrEP drugs (3‐month supply)*	−	76 (76%); $8.21 ($4.11−$21.43)	529 (83%); $10.71 ($6.43 ‐ $14.29)
*PEP drugs (28‐day course)*	−	82 (82%); $3.57 ($2.14−$7.14)	507 (80%); $3.57 ($2.14 ‐ $7.14)

Abbreviations: CSQ, Client Satisfaction Questionnaire; HIVST, HIV self‐testing; IQR, interquartile range; ‐, not applicable; PEP, post‐exposure prophylaxis; PrEP, pre‐exposure prophylaxis; SD, standard deviation; TFA, Theoretical Framework of Acceptability; USD, United States Dollar.

^a^ Questions asked only to subset of survey participants who initiated online PrEP and PEP.

^b^ Response options listed here are the Likert‐scale outcomes that were grouped together; for more detailed responses, see Table S3.

^c^ The Theoretical Framework of Acceptability (TFA) defines acceptability as a multi‐faceted construct, made up of several different component constructs.

^d^ Client Satisfaction Questionnaire‐8 (CSQ‐8) is an 8‐item standardized tool used to assess client satisfaction with services. The CSQ‐8 assesses satisfaction on each of the 8 items with a 4‐point scale and provides a general score ranging from 8 to 32 (with higher points equating to greater satisfaction).

^e^ Among participants who ordered an HIVST (PrEP clients: 94%, 94/100; PEP clients: 92%, 585/638) or RDT (PrEP clients: 6%, 6/100; PEP clients: 8%, 49/638).

^f^ Average USD/KSH 2023 exchange rate = 140 KSH per 1 USD. The median reported willingness to pay is among those who reported they were willing to pay something.

## 4 DISCUSSION

Online PrEP/PEP delivery reached PrEP/PEP naïve individuals at HIV acquisition risk [40] and online PEP services were particularly in demand, with some clients returning for repeat PEP and few clients transitioning from PEP to PrEP. Online PrEP continuation was low, but still comparable to rates observed at Kenyan public clinics [41, 42, 43, 44, 45, 46]. Taken together, these findings highlight a demand for periodic versus persistent use of online HIV prevention services. Additionally, online PrEP/PEP clients found the model acceptable, satisfactory and high‐quality, and many were willing to pay for online PrEP/PEP—suggesting the sustainability of the model at scale. Further, almost all online PEP clients received drugs within 72 hours of a potential HIV exposure, demonstrating the ability of the model to reach clients with time‐sensitive needs. These findings suggest the feasibility of a public‐private partnership model that facilitates delivery of government HIV commodities on a for‐profit e‐commerce platform in Kenya.

In our pilot, the uptake of online PEP was five times greater than online PrEP. This underscores the unmet demand for PEP and the need for widespread PEP availability and interventions catered to periodic HIV prevention services (e.g. event‐driven PrEP). Despite considerable PrEP scale‐up efforts in Kenya, implementation challenges persist, including low PrEP uptake and continuation at public clinics (<35% at 6 months) [43, 44, 45, 46]. The greater observed demand for PEP versus PrEP in this pilot is difficult to compare with that at Kenyan public clinics, as PEP dispensing is not documented in Kenya's clinic‐based electronic reporting tool for antiretroviral drug dispensing. The few implementation projects in Kenya that have offered PrEP and PEP as equal HIV prevention options also observed high PEP uptake; in two implementation projects delivering PrEP/PEP in brick‐and‐mortar private pharmacies, 20% and 68% of clients initiated PEP over PrEP [47, 48], and in an implementation project delivering PrEP/PEP in the community, 58% of clients initiated PEP over PrEP [49].

The greater observed uptake of PEP versus PrEP in this pilot may have several contributing factors. First, the risk of a potential HIV exposure has already occurred for PEP clients but is hypothetical for many PrEP clients. Thus, PEP clients are seeking time‐sensitive services (and potentially searching the internet for assistance), while potential PrEP clients may need prompting to consider prevention for future HIV risk (a conversation that is hard to initiate online). Second, community‐level PEP awareness in Nairobi and Mombasa Counties might be higher than PrEP since PEP has been around longer and most PrEP programming in Kenya has targeted the Western region, where HIV prevalence is greatest. Third, extended operating hours of online pharmacies (including evenings and weekends) might better align with when potential PEP clients seek this time‐sensitive service. Fourth, the speed and convenience with which online pharmacies can deliver services (≤6 hours) might be appealing to PEP clients with urgent prevention needs.

Online PrEP/PEP delivery may expand the reach of HIV prevention products to those not accessing clinic‐based services. We found most online PrEP/PEP clients were PrEP and PEP naïve, unmarried, male and not in serodifferent relationships, whereas clients accessing PrEP at public clinics are largely married, female and in serodifferent relationships [42, 43]. Importantly, online PrEP reached many men reporting sex with men, highlighting the potential of online services to reach populations less likely to seek in‐person services. Additionally, most online PEP clients reported a potential recent HIV exposure through consensual condomless sex versus sexual assault or exposure to a non‐sexual bodily fluid, the latter two of which have been the focus of most PEP programing in the region. Most online PrEP/PEP clients also preferred HIVST over provider‐administered HIV testing, highlighting the importance of privacy and autonomy during the testing process.

Strengths of this study include collaboration of a multidisciplinary team of researchers, implementors, entrepreneurs and government officials; integration of AI to help ensure service quality; and assessment of implementation and willingness to pay outcomes. Limitations include utilizing subsidized HIVST kits and free telehealth visits, which may limit model sustainability; charging for products, which may have altered demand; changes in implementation midway through observation (i.e. delivery fees or HIV testing options), due to collaboration with a real‐world e‐commerce platform; relatively low participation in behavioural surveys, which may have biased findings towards positive perceptions; exclusion of event‐driven PrEP; and insufficient information regarding how soon clients took their first PEP dose following courier delivery. Further, online PrEP/PEP delivery largely reached clients with high education, ability to pay for HIV testing and internet access/literacy; more research is needed to evaluate telehealth modalities to serve those with lower socio‐economic status who could benefit from PrEP/PEP.

To optimize online PrEP/PEP delivery and support its scale‐up in Kenya and similar settings, various implementation strategies could be evaluated. Integration of digital adherence support strategies—including personalized SMS messages [50], AI assistants [51] or digital rewards (i.e. badges) [52]—could improve follow‐up. Allowing clients to schedule deliveries at specific times or pick up products at select locations (e.g. brick‐and‐mortar pharmacies) could improve privacy and convenience. Facilitating insurance coverage of online PrEP/PEP and distributing vouchers or discount codes [53] could decrease access barriers for clients unable to pay (e.g. adolescent girls and young women). Pharm tech couriers could be replaced with lower cadre healthcare workers—such as HIV testing service counsellors—to lower implementation costs. Additionally, remote clinicians could be leveraged for prescribing other medications to generate additional revenue. Finally, the development of automated reporting tools and integration of Kenya's electronic clinical records for antiretroviral dispensing into the online PrEP/PEP delivery platform could facilitate the distribution of public commodities at private online pharmacies.

## 5 CONCLUSIONS

Our findings underscore the potential for online pharmacies to expand PrEP/PEP coverage to eligible clients not accessing clinic‐based prevention services. The high PEP uptake, repeat PEP use and low PEP‐to‐PrEP transition suggest a high unmet demand for periodic HIV prevention services among online pharmacy clients. Our findings emphasize the need for choice in HIV prevention options, the importance of providing PEP as part of comprehensive HIV services and the role online pharmacies could play in the delivery of time‐sensitive PEP.

## COMPETING INTERESTS

The authors declare no conflicts of interest.

## AUTHORS’ CONTRIBUTIONS

KFO, MS, MLM, KN and DW contributed to the study conception and design of this pilot study. MR, SA, EJ and NN led recruitment and implementation operations with support from CK, TK, NT, DW and JM. KFO and MS designed the analysis plan and PN analysed the data. CK, PN, KFO and MS wrote the first draft of this manuscript. All authors edited the draft, provided insights and approved the final manuscript for publication.

## FUNDING

This work was supported by the Bill and Melinda Gates Foundation (INV‐037646). KFO and MS both received additional funding from the National Institute of Mental Health (R00 MH121166; K01 MH115789).

## Supporting information




**Figure S1**. Care pathway for delivery of online PrEP/PEP services

**Figure S2**. Example of social media advertisement for MYDAWA's online PrEP/PEP services

**Figure S3**. Prescribing checklist for online PrEP/PEP service delivery

**Figure S4**. How online PrEP/PEP clients learned of online PrEP/PEP services

**Table S1**. Characteristics associated with PrEP continuation among online PrEP clients—bivariable regression outputs

**Table S2**. Characteristics associated with PEP‐to‐PrEP transition and repeat PEP use among online PEP clients—bivariable regression outputs

**Table S3**. Details on clients’ perceived acceptability of, experiences with and quality of online PrEP/PEP services

**Table S4**. Differences in client characteristics between those who did and did not complete the behavioural surveys

## Data Availability

The data from this pilot study are available on request from the corresponding author and not publicly available due to privacy and ethical restrictions.
