# Supplementary figures and images for: Online delivery of oral HIV pre‐ and post‐exposure prophylaxis: findings from the ePrEP Kenya pilot
Source: J Int AIDS Soc. 2025 Jun 26;28(Suppl 1):e26468. doi: 10.1002/jia2.26468 (PMC12231658; doi:10.1002/jia2.26468)

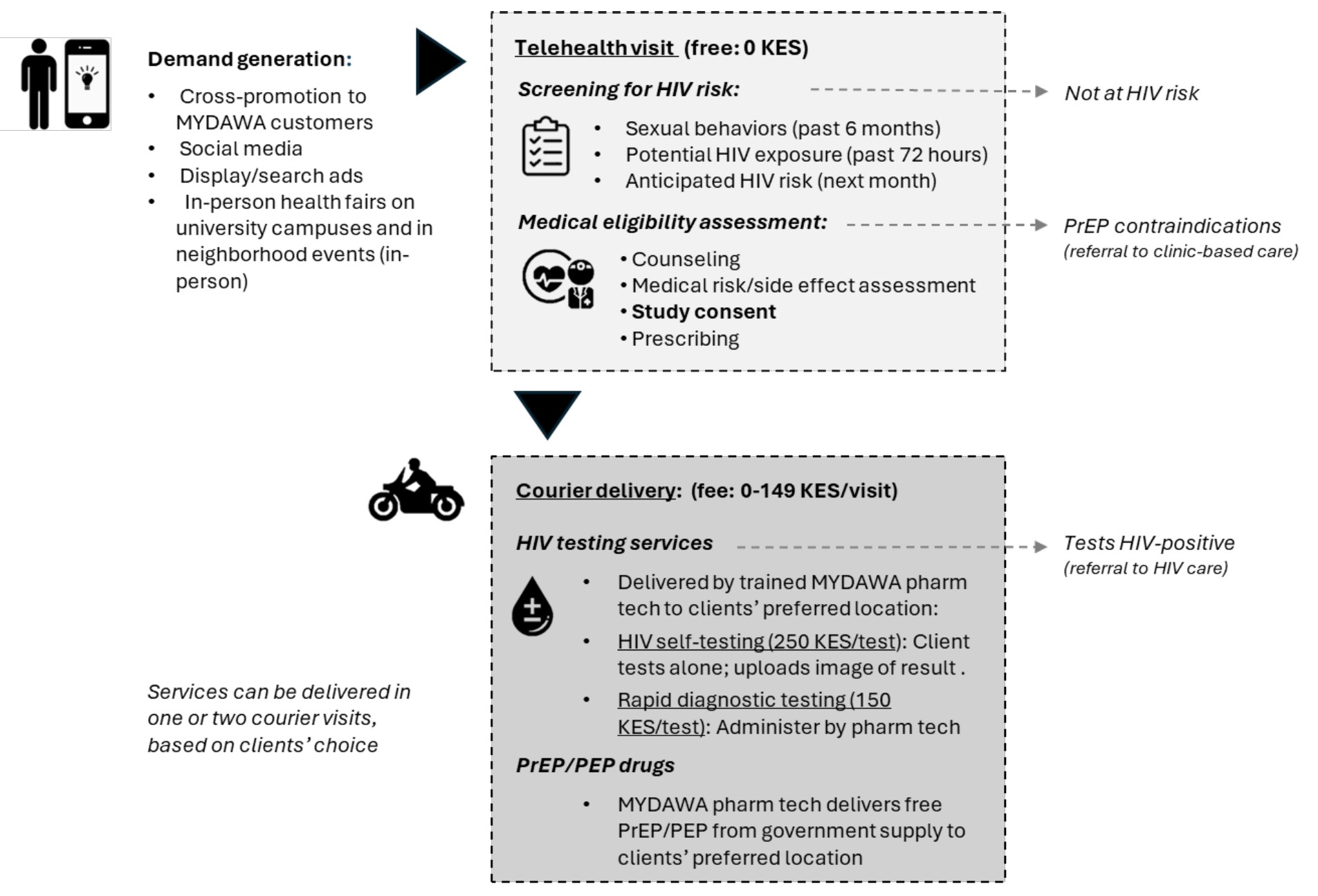

Supplement: Supplementary file 1 — Figure S1. Care pathway for delivery of online PrEP/PEP services [file JIA2-28-e26468-s008.jpg]

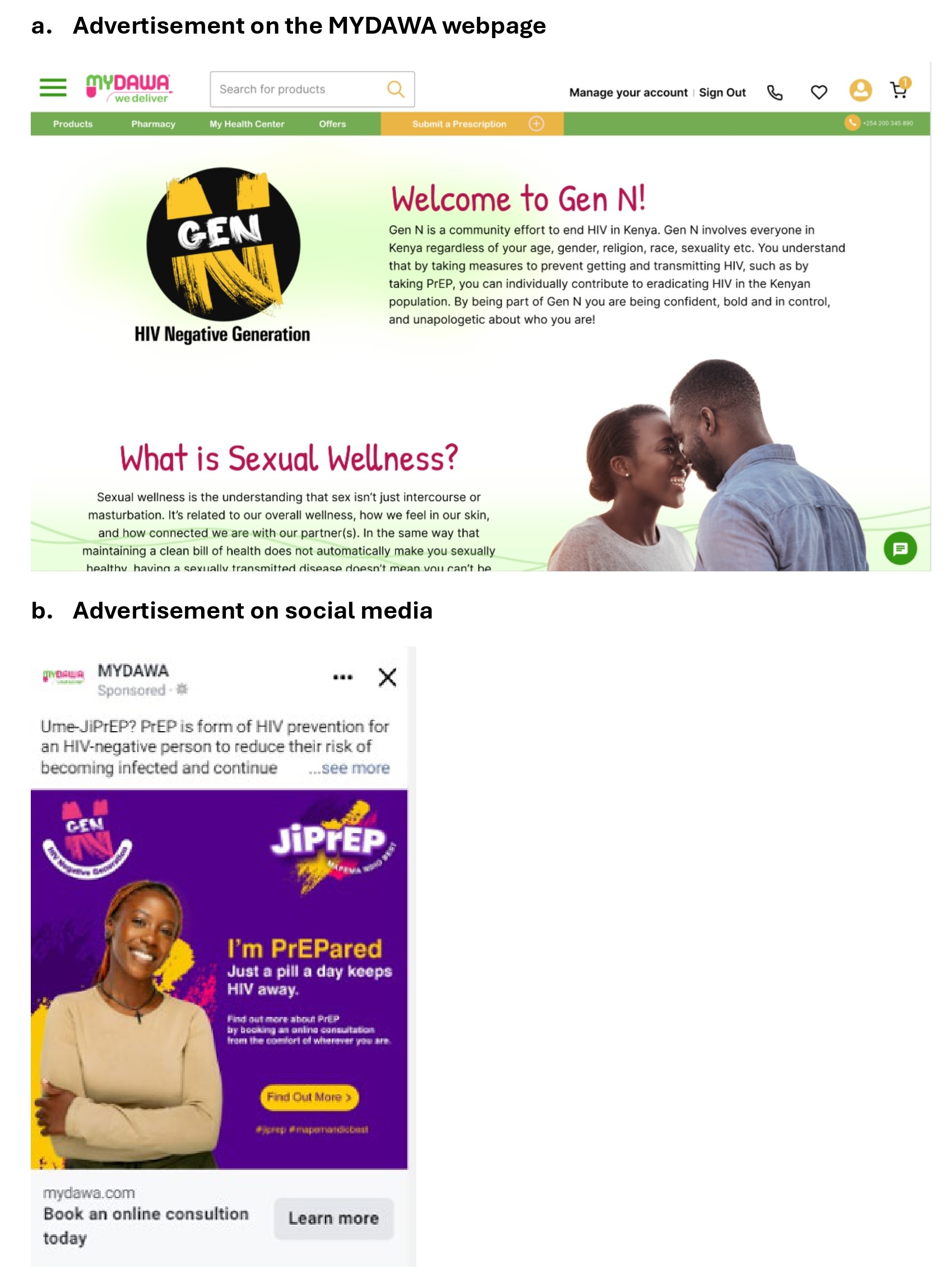

Supplement: Supplementary file 2 — Figure S2. Example of social media advertisement for MYDAWA's online PrEP/PEP services [file JIA2-28-e26468-s003.jpg]

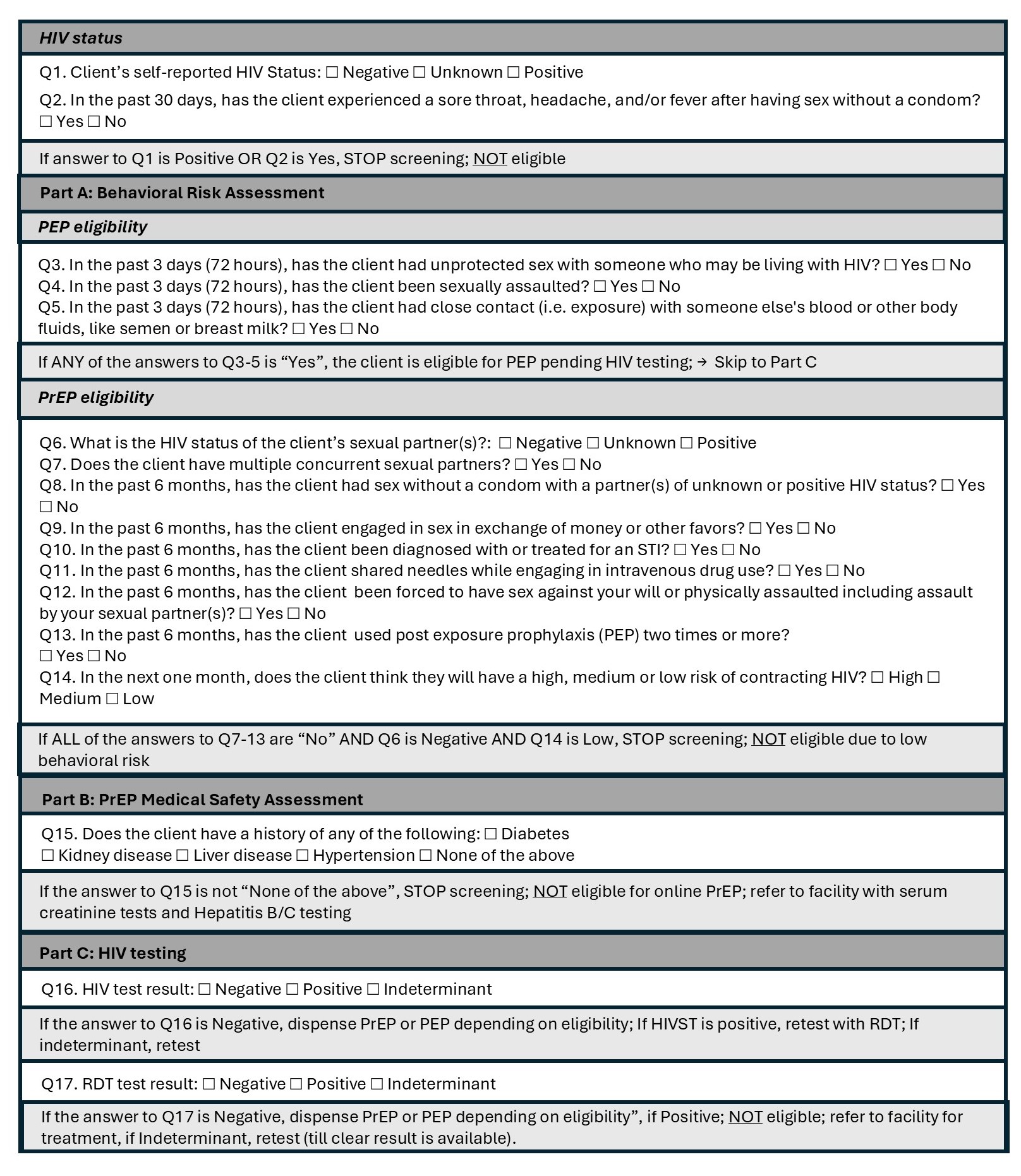

Supplement: Supplementary file 3 — Figure S3. Prescribing checklist for online PrEP/PEP service delivery [file JIA2-28-e26468-s005.jpg]

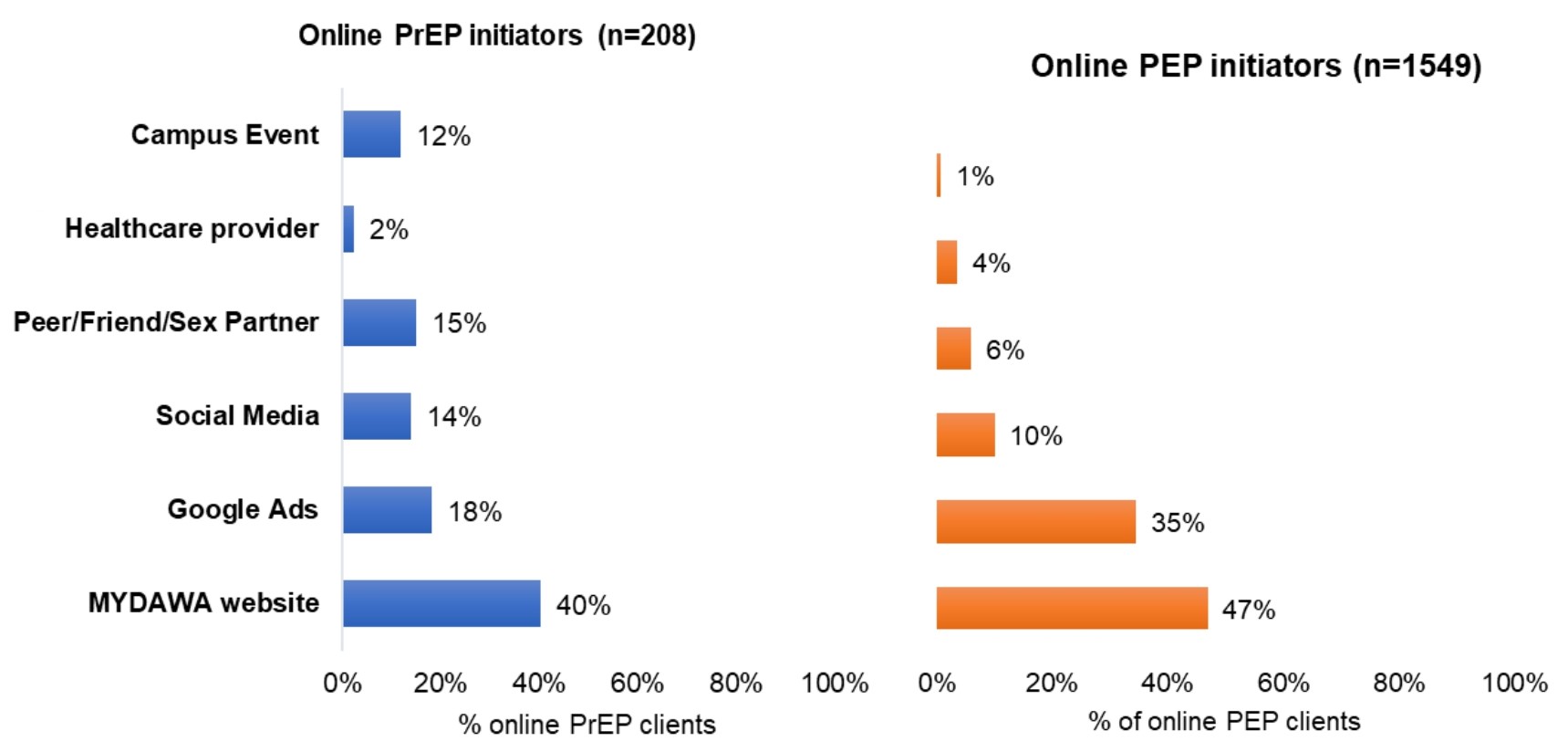

Supplement: Supplementary file 4 — Figure S4. How online PrEP/PEP clients learned of online PrEP/PEP services [file JIA2-28-e26468-s001.jpg]
