# Supplementary material for: Online delivery of oral HIV pre‐ and post‐exposure prophylaxis: findings from the ePrEP Kenya pilot
Source: J Int AIDS Soc. 2025 Jun 26;28(Suppl 1):e26468. doi: 10.1002/jia2.26468 (PMC12231658; doi:10.1002/jia2.26468)
Supplement: Supplementary file 5 — Table S1. Characteristics associated with PrEP continuation among online PrEP clients—bivariable regression outputs [file JIA2-28-e26468-s002.pdf]

Table S1. Characteristics associated with PrEP continuation among online PrEP clients—bivariable regression outputs

Characteristic	Initiated PrEP, n=256 †			
	**Refilled PrEP (n=88)**	**Did not refill PrEP (n=168)**	**RR (95% CI)**	**p-value**
**Demographics**				
Age ≥ 25 years	**75 (85%)**	**114 (69%)**	**1.98 (1.18-3.33)**	**0.01**
Sex: Male	64 (73%)	118 (70%)	1.08 (0.74-1.59)	0.68
Married	8 (9%)	16 (10%)	0.95 (0.53-1.73)	0.88
Men who have sex with men	**21 (24%)**	**21 (13%)**	**1.60 (1.11-2.29)**	**0.01**
**Health history**				
Prior PrEP use ‡	**17 (19%)**	**17 (10%)**	**1.54 (1.05-2.27)**	**0.03**
Prior PEP use ‡	14 (16%)	45 (27%)	0.62 (0.38 -1.02)	0.06
Forced to have sex §	0 (0%)	3 (2%)	-	-
STI diagnosis §	2 (2%)	6 (4%)	0.72 (0.21-2.42)	0.60
**Sexual Behaviors**				
1+ sex partner	58 (66%)	93 (56%)	1.33 (0.93-1.91)	0.12
Partner(s) unknown HIV status	64 (74%)	134 (80%)	0.80 (0.55-1.16)	0.25
Partner living with HIV	**12 (14%)**	**9 (5%)**	**1.78 (1.18-1.04)**	**0.01**
Inconsistent condom use	47 (53%)	96 (57%)	0.91 (0.65-1.27)	0.57
Transactional sex	2 (2%)	4 (2%)	0.97 (0.31-3.04)	0.96
Unprotected sex (past 72 hours)	0 (0%)	11 (7%)	-	-
Exposure to bodily fluids (past 72 hours)	2 (2%)	0 (0%)	-	-
**Self-assessed HIV risk, next month**				
Low (Ref.)	13 (15%)	29 (17%)	Ref	
Medium	40 (45%)	78 (47%)	0.72 (0.49-1.09)	0.11
High	35 (40%)	60 (36%)	0.97 (0.55- 1.67)	0.92

Abbreviations: Risk ratio (RR); p-value (p); pre-exposure prophylaxis (PrEP); post-exposure prophylaxis (PEP); sexually-transmitted infection (STI).

† Among those eligible for follow up

‡ At the time of initial consultation

§ In the past 6 months at the time of consultation

- Insufficient observations for negative binomial regression model
