# Supplementary material for: Online delivery of oral HIV pre‐ and post‐exposure prophylaxis: findings from the ePrEP Kenya pilot
Source: J Int AIDS Soc. 2025 Jun 26;28(Suppl 1):e26468. doi: 10.1002/jia2.26468 (PMC12231658; doi:10.1002/jia2.26468)
Supplement: Supplementary file 6 — Table S2. Characteristics associated with PEP‐to‐PrEP transition and repeat PEP use among online PEP clients—bivariable regression outputs [file JIA2-28-e26468-s006.pdf]

Table S2. Characteristics associated with PEP-to-PrEP transition and repeat PEP use among online PEP clients—bivariable regression outputs

Characteristic	Initiated PEP (n=1428)[†]							
	Transitioned to PrEP (n=82)	Did not transition to PrEP (n=1346)	PR (95% CI)	p	Repeated PEP (n=99)	Did not repeat PEP (n=1329)	PR (95% CI)	p
**Demographics**								
Age ≥ 25 years	57 (70%)	962 (72%)	0.89 (0.56-1.40)	0.62	66 (67%)	953 (72%)	0.78 (0.52-1.17)	0.23
Sex: Male	53 (65%)	838 (62%)	1.10 (0.71-1.71)	0.67	**75 (76%)**	**816 (61%)**	**1.88 (1.20-2.94)**	**0.01**
Married	**1 (1%)**	**174 (13%)**	**0.09 (0.01-0.63)**	**0.02**	8 (8%)	167 (13%)	0.63 (0.31-1.27)	0.19
Men who have sex with men	**8 (10%)**	**16 (1%)**	**6.32 (3.44- 11.61)**	**<0.001**	3 (3%)	21 (2%)	1.83 (0.62-5.36)	0.27
**Health history**								
Prior PrEP use [‡]	**3 (4%)**	**8 (1%)**	**4.91 (1.83- 13.12)**	**<0.001**	10 (1%)	1 (1%)	1.30 (0.20-8.52)	0.78
Prior PEP use [‡]	**20 (25%)**	**175 (13%)**	**2.07 (1.28- 3.35)**	**0.003**	**26 (26%)**	**169 (13%)**	**2.21 (1.45-3.37)**	**<0.001**
Forced to have sex [§]	1 (1%)	35 (3%)	0.48 (0.07- 3.35)	0.46	3 (3%)	33 (3%)	1.21 (0.40-3.64)	0.73
STI diagnosis [§]	5 (6%)	35 (3%)	2.24 (0.96- 5.22)	0.06	3 (3%)	37 (3%)	1.10 (0.36-3.32)	0.87
**Sexual Behaviors**								
1+ sex partner	36 (44%)	610 (46%)	0.93 (0.61-1.42)	0.73	41 (41%)	605 (46%)	0.83 (0.57-1.23)	0.36
Partner(s) unknown HIV status	75 (94%)	1204 (90%)	1.64 (0.68- 3.99)	0.27	88 (90%)	1191 (90%)	0.96 (0.51-1.81)	0.91
Partner living with HIV	3 (4%)	36 (3%)	1.38 (0.45- 4.18)	0.57	5 (5%)	34 (3%)	1.90 (0.82-4.41)	0.13
Inconsistent condom use	65 (79%)	992 (74%)	1.32 (0.78- 2.22)	0.30	**64 (66%)**	**993 (75%)**	**0.67 (0.45-1.00)**	**0.05**
Transactional sex	4 (5%)	33 (3%)	1.92 (0.74- 4.96)	0.18	3 (3%)	34 (3%)	1.18 (0.39-3.55)	0.77
Unprotected sex (past 72 hours)	65 (79%)	986 (74%)	1.32 (0.79- 2.23)	0.29	75 (78%)	976 (74%)	1.24 (0.77 -1.98)	0.37
Exposure to bodily fluids (past 72 hours)	15 (18%)	256 (19%)	0.95 (0.55- 1.64)	0.85	13 (13%)	258 (20%)	0.66 (0.37 -1.16)	0.15
**Self-assessed HIV risk, next month**								
Low (Ref.)	16 (20%)	326 (24%)	Ref		19 (19%)	323 (24%)	Ref	
Medium	57 (70%)	831 (62%)	1.39 (0.81- 2.39)	0.23	67 (68%)	823 (62%)	1.36 (0.83-2.22)	0.23
High	8 (10%)	186 (14%)	0.88 (0.38- 2.02)	0.77	12 (12%)	182 (14%)	1.11 (0.55-2.24)	0.76

Abbreviations: percentile rank (PR); confidence interval (CI); p-value (p); pre-exposure prophylaxis (PrEP); post-exposure prophylaxis (PEP); sexually-transmitted infection.

[†] Among those eligible for follow up

[‡] At the time of initial consultation

[§] In the past 6 months at the time of consultation
