# Supplementary material for: Online delivery of oral HIV pre‐ and post‐exposure prophylaxis: findings from the ePrEP Kenya pilot
Source: J Int AIDS Soc. 2025 Jun 26;28(Suppl 1):e26468. doi: 10.1002/jia2.26468 (PMC12231658; doi:10.1002/jia2.26468)
Supplement: Supplementary file 7 — Table S3. Details on clients’ perceived acceptability of, experiences with and quality of online PrEP/PEP services [file JIA2-28-e26468-s007.pdf]

Table S3. Details on clients' perceived acceptability of, experiences with, and quality of online PrEP/PEP services †

**Acceptability**	**Completely disagree**	**Disagree**	**Neutral**	**Agree**	**Completely agree**
**PrEP clients, n=93**					
Liked getting PrEP online (TFA: affective attitude)	0 (0%)	0 (0%)	0 (0%)	4 (4%)	87 (94%)
Took a lot of effort to get PrEP online (TFA: burden)	43 (46%)	27 (29%)	4 (4%)	5 (5%)	10 (11%)
Confident in ability to get PrEP online (TFA: self-efficacy)	2 (2%)	0 (0%)	2 (2%)	6 (7%)	81 (87%)
Getting PrEP online helps prevent the spread of HIV (TFA: perceived effectiveness)	0 (0%)	1 (1%)	7 (8%)	15 (16%)	69 (74%)
Getting PrEP online was acceptable	0 (0%)	0 (0%)	1 (1%)	8 (9%)	81 (87%)
**PEP clients, n=638**					
Liked getting PEP online (TFA: affective attitude)	6 (1%)	0 (0%)	7 (1%)	39 (6%)	575 (89%)
Took a lot of effort to get PEP online (TFA: burden)	268 (42%)	216 (34%)	39 (6%)	38 (6%)	66 (10%)
Confident in ability to get PEP online (TFA: self-efficacy)	3 (0.5%)	2 (0.3%)	2 (0.3%)	44 (7%)	576 (90%)
Getting PEP online can help prevent the spread of HIV (TFA: perceived effectiveness)	2 (0.3%)	22 (3%)	30 (5%)	101 (16%)	471 (73%)
Getting PEP online was acceptable	0 (0%)	1 (0.2%)	3 (0.5%)	52 (8%)	568 (88%)
**Experiences with intervention**	**Extremely negative**	**Negative**	**Neutral**	**Positive**	**Extremely positive**
**PrEP clients, n=93**					
Learning about PrEP/PEP on MYDAWA's "My Health Center" page	0 (0%)	0 (0%)	2 (2%)	25 (27%)	53 (57%)
Consulting remote clinician for PrEP/PEP prescription	0 (0%)	0 (0%)	0 (0%)	28 (30%)	60 (65%)
Ordering HIVST from MYDAWA ‡ (/ HIVST users)	0 (0%)	0 (0%)	4 (4%)	28 (30%)	44 (47%)
Getting HIVST delivered from MYDAWA ‡ (/ HIVST users)	1 (1%)	0 (0%)	2 (2%)	26 (28%)	47 (51%)
Uploading HIVST result image to MYDAWA ‡ (/ HIVST users)	0 (0%)	0 (0%)	10 (11%)	35 (40%)	38 (41%)
Having RDT administered by a MYDAWA clinician ‡ (/ RDT users)	0 (0%)	0 (0%)	0 (0%)	1 (17%)	5 (83%)
Getting PrEP delivered from MYDAWA	0 (0%)	0 (0%)	5 (5%)	27 (29%)	54 (58%)
**PEP clients, n=638**					
Learning about PrEP/PEP on MYDAWA's "My Health Center" page	2 (0.3%)	0 (0%)	6 (1%)	186 (29%)	364 (57%)
Consulting remote clinician for PrEP/PEP prescription	1 (0.2%)	2 (0.3%)	14 (2%)	215 (34%)	402 (63%)
Ordering HIVST from MYDAWA ‡ (/ HIVST users)	0 (0%)	6 (1%)	17 (3%)	257 (43%)	295 (50%)
Getting HIVST delivered from MYDAWA ‡ (/ HIVST users)	1 (0.2%)	8 (1%)	26 (4%)	225 (38%)	317 (53%)
Uploading HIVST result image to MYDAWA ‡ (/ HIVST users)	1 (0.2%)	23 (4%)	63 (11%)	259 (44%)	233 (39%)
Having RDT administered by a MYDAWA clinician ‡ (/ RDT users)	0 (0%)	0 (0%)	0 (0%)	12 (24%)	37 (75%)
Getting PEP delivered from MYDAWA	4 (1%)	7 (1%)	28 (4%)	227 (35%)	366 (57%)
**Quality of care received**	**Strongly disagree**	**Disagree**	**Neutral**	**Agree**	**Strongly agree**
**PrEP clients, n=93**					
Connection for remote consultation was stable	0 (0%)	1 (1%)	2 (2%)	12 (13%)	71 (77%)
MYDAWA clinician used language that was easy to understand	1 (1%)	0 (0%)	-	7 (8%)	85 (91%)
MYDAWA clinician acted judgmental	78 (84%)	11 (12%)	-	0 (0%)	4 (4%)
MYDAWA clinician encouraged questions	1 (1%)	2 (2%)	-	5 (5%)	85 (91%)
MYDAWA clinician was respectful	1 (1%)	0 (0%)	-	4 (4%)	88 (95%)
MYDAWA clinician listened without interrupting	0 (0%)	0 (0%)	-	7 (8%)	86 (93%)
Participant willing to seek help from same clinician again	1 (1%)	3 (3%)	-	6 (7%)	81 (87%)
**PEP clients, n=638**					
Connection for remote consultation was stable	2 (0.3%)	4 (1%)	16 (3%)	100 (16%)	508 (79%)
MYDAWA clinician used language that was easy to understand	4 (1%)	0 (0%)	-	33 (5%)	598 (93%)
MYDAWA clinician acted judgmental	497 (77%)	104 (16%)	-	4 (1%)	31 (5%)
MYDAWA clinician encouraged questions	4 (1%)	3 (1%)	-	48 (8%)	578 (90%)
MYDAWA clinician was respectful	2 (0.3%)	0 (0%)	-	22 (4%)	609 (95%)
MYDAWA clinician listened without interrupting	2 (0.3%)	0 (0%)	-	27 (4%)	602 (94%)

Participant willing to seek help from same clinician again	1 (0.2%)	3 (1%)	-	23 (4%)	606 (94%)

Abbreviations: Theoretical Framework of Acceptability (TFA).

† Percentages across each row may not add up to 100 due to missing data.

‡ Among participants that ordered an HIVST (PrEP clients: 94%, 94/100; PEP clients: 92%, 585/638) or RDT (PrEP clients: 6%, 6/93; PEP clients: 8%, 49/638).
