# Supplementary material for: Online delivery of oral HIV pre‐ and post‐exposure prophylaxis: findings from the ePrEP Kenya pilot
Source: J Int AIDS Soc. 2025 Jun 26;28(Suppl 1):e26468. doi: 10.1002/jia2.26468 (PMC12231658; doi:10.1002/jia2.26468)
Supplement: Supplementary file 8 — Table S4. Differences in client characteristics between those who did and did not complete the behavioural surveys [file JIA2-28-e26468-s004.pdf]

Table S4. Differences in client characteristics between those who did and did not complete the behavioral surveys

Characteristic	Preliminarily eligible online PrEP clients (n=227)			Preliminarily eligible online PEP clients (n=1688)		
	Completed survey (n=93)	Did not complete survey (n=134)	p-value	Completed survey (n=638)	Did not complete survey (n=1050)	p-value
**Demographics**						
Age ≥25 years	59 (63%)	100 (75%)	0.07	434 (68%)	776 (74%)	**0.01**
Sex: *Male* †	57 (61%)	111 (83%)	**<0.01**	392 (61%)	674 (64%)	0.3
Married	5 (5%)	21 (16%)	0.03	79 (12%)	133 (13%)	0.9
Special populations						
Men who have sex with men (/men)	15 (16%)	32 (24%)	0.2	13 (2%)	15 (1.4%)	0.5
Member of HIV serodiscordant couple	3 (3%)	7 (5%)	0.7	0 (%)	1 (0.1%)	1
**Health history** ‡						
Currently using PrEP	6 (7%)	16 (12%)	0.3	0 (0%)	0 (0%)	-
Prior PrEP use	13 (14%)	23 (17%)	0.6	5 (1%)	7 (1%)	1
Prior PEP use	15 (16%)	21 (16%)	1	100 (16%)	127 (12%)	0.04
Pregnant/breastfeeding (/women)	2 (6%)	0 (0%)	0.3	2 (1%)	5 (1%)	0.9
**Behaviors associated with risk of HIV acquisition** ‡						
In the past 6 months						
Multiple concurrent sexual partners	56 (60%)	88 (66%)	0.5	281 (44%)	508 (48%)	0.1
Partner(s) of unknown HIV status	72 (78%)	98 (73%)	0.6	569 (89%)	938 (89%)	1
Partner(s) living with HIV	9 (10%)	16 (12%)	0.7	16 (3%)	27 (3%)	1
Inconsistent condom use	45 (48%)	67 (50%)	0.9	450 (71%)	781 (74%)	1
Transactional sex	2 (2%)	4 (3%)	1	19 (3%)	26 (3%)	0.6
STI diagnosis	4 (4%)	3 (2%)	0.6	20 (3%)	27 (3%)	0.6
Needle sharing for drug use	0 (0%)	0 (0%)	-	0 (0%)	4 (0%)	0.3
Forced sex/sexual assault	1 (1%)	4 (3%)	0.6	16 (3%)	22 (2%)	0.3
Used PEP 2+ times	5 (5%)	8 (6%)	1	24 (4%)	38 (4%)	1
In the past 72 hours ‡						
Unprotected sex and potential risk of HIV acquisition	5 (5%)	2 (2%)	0.2	468 (73%)	733 (70%)	0.1
Sexual assault	0 (0%)	0 (0%)	-	3 (1%)	14 (1%)	0.1
Exposure to bodily fluids: non-sexual	0 (0%)	0 (0%)	-	23 (4%)	47 (5%)	0.5
Exposure to bodily fluids: sexual	1 (1%)	0 (0%)	0.8	138 (22%)	222 (21%)	0.9
Self-assessment of HIV risk, next month						
High	23 (25%)	58 (43%)	**0.01**	81 (13%)	144 (14%)	0.3
Medium	48 (52%)	55(41%)		403 (63%)	624 (59%)	
Low	22 (24%)	20 (15%)		152 (24%)	276 (26%)	

Abbreviations: pre-exposure prophylaxis (PrEP); post-exposure prophylaxis (PEP); sexually-transmitted infection (STI).

† This includes two PrEP clients who identified as intersex (included in all PrEP categories) and one PEP client who identified as intersex; this individual did not participate in the behavioral survey.

‡ Categories are not mutually exclusive.
